# The Role of Ku70 as a Cytosolic DNA Sensor in Innate Immunity and Beyond

**DOI:** 10.3389/fcimb.2021.761983

**Published:** 2021-10-21

**Authors:** Hongyan Sui, Ming Hao, Weizhong Chang, Tomozumi Imamichi

**Affiliations:** Laboratory of Human Retrovirology and Immunoinformatics, Frederick National Laboratory for Cancer Research, Frederick, MD, United States

**Keywords:** Ku70, Ku heterodimer, cytosolic DNA sensing, innate immunity, interferons, HIV replication

## Abstract

Human Ku70 is a well-known endogenous nuclear protein involved in the non-homologous end joining pathway to repair double-stranded breaks in DNA. However, Ku70 has been studied in multiple contexts and grown into a multifunctional protein. In addition to the extensive functional study of Ku70 in DNA repair process, many studies have emphasized the role of Ku70 in various other cellular processes, including apoptosis, aging, and HIV replication. In this review, we focus on discussing the role of Ku70 in inducing interferons and proinflammatory cytokines as a cytosolic DNA sensor. We explored the unique structure of Ku70 binding with DNA; illustrated, with evidence, how Ku70, as a nuclear protein, responds to extracellular DNA stimulation; and summarized the mechanisms of the Ku70-involved innate immune response pathway. Finally, we discussed several new strategies to modulate Ku70-mediated innate immune response and highlighted some potential physiological insights based on the role of Ku70 in innate immunity.

## Introduction

Innate immunity includes diverse areas of host defense response to pathogen invasion, such as bacterial or viral infection. In this system, pattern recognition receptors (PRRs) expressed in host cells recognize the conserved pathogen-associated molecular patterns (PAMPs) which are derived from microbes and then mediate innate immune responses (Medzhitov and Janeway, 1997; Medzhitov and Janeway, 2000; Akira et al., 2006; Pichlmair and Reis e Sousa, 2007; Mogensen, 2009; Takeuchi and Akira, 2009; Yoneyama and Fujita, 2010). Detection of pathogenic cytosolic nucleic acids: double-stranded (ds) or single-stranded (ss) DNA and RNA is essential to initiate innate immunity. PRR families include the retinoic acid-inducible gene I (RIG-I)-like receptors, toll-like receptors (TLRs), and a diverse member of cytosolic DNA sensors (Bowie and Haga, 2005; Kaisho and Akira, 2006; Yoneyama and Fujita, 2008; Beutler, 2009; Kawai and Akira, 2009; Yoneyama and Fujita, 2009; Barber, 2011; Keating et al., 2011; Thompson et al., 2011; Paludan and Bowie, 2013; Dempsey and Bowie, 2015). Once PAMPs are sensed by PRRs, the recognition subsequently mediates intracellular signaling pathways and activates transcription factors, interferon (IFN) regulatory factors (IRFs) or nuclear factor κB (NF-κB), which in turn leads to the increased production of antiviral interferons and proinflammatory cytokines (Lee and Kim, 2007; Mogensen, 2009).

DNA is a potent trigger of innate immune responses in host cells (Sharma and Fitzgerald, 2011). Many studies have emphasized the importance of cytosolic DNA sensing in the innate immune response against invading pathogens. The DNA-mediated innate immune response includes diverse signaling pathways leading to the production of IFN-α, IFN-β, interleukin (IL)-1β, or IL-18 (Christensen and Paludan, 2016). For instance, the DNA-dependent activator of IFN-regulatory factors (DAI) (Takaoka, 2007) is the first identified DNA sensor to recognize dsDNA and activate the STING-TBK1-IRF3 signaling pathway. After that, gamma-interferon-inducible protein (IFI16) (Unterholzner et al., 2010; Monroe et al., 2014; Thompson et al., 2014) and DEAD-box helicase 41 (DDX41) (Zhang et al., 2011c) were found as cytosolic DNA sensors in diverse cellular processes to recognize DNA. Leucine-rich repeat flightless-interacting protein 1 (LRRFIP1), another discovered cytosolic DNA sensor, binds dsDNA and activates β-catenin to induce downstream signaling (Yang et al., 2010). DEAH box protein 9 (DHX9) and DHX36 bind with dsDNA in dendritic cells and activate NF-κB through myeloid differentiation primary response 88 (MyD88) (Kim et al., 2010; Zhang et al., 2011b). More recently, cyclic GMP-AMP Synthase (cGAS) has been identified as a cytosolic DNA sensor (Gao et al., 2013; Sun et al., 2013; Cai et al., 2014; Zhang et al., 2014; Xia et al., 2016). Once cGAS detects dsDNA, it undergoes a conformational change to open the catalytic pocket followed by synthesis of cGAMP from ATP and GTP: a potent activator of the STING-TBK1-IRF3 signaling pathway. In addition to the induction pathway of IFNs, Absent in melanoma 2 (AIM2) has been found to associate with cytosolic DNA and activate inflammasomes by recruiting apoptosis-associated speck-like protein (ASC) and pro-caspase-1, and then produce mature forms of IL-1β and IL-18 (Burckstuummer, 2009; Fernandes-Alnemri et al., 2009; Hornung, 2009).

The DNA-mediated innate immune response is not restricted to the induction of type I IFNs and proinflammatory cytokines: cytosolic DNA also induces type III IFNs. Type III IFNs are new members of the IFN family (Ank et al., 2006; Ank and Paludan, 2009; Donnelly and Kotenko, 2010; Kotenko, 2011; Syedbasha and Egli, 2017). Type III IFNs are also called IFN-λs, which include IFN-λ1, IFN-λ2, IFN-λ3 (also known as IL-29, IL-28A, and IL-28B, respectively) and IFN-λ4 (Ank et al., 2006; Uzé and Monneron, 2007; Ank and Paludan, 2009; Kotenko, 2011; Booth and George, 2013; Lu et al., 2015). Compared with type I IFNs, they use a different heterodimeric receptor complex (IFN-λR1/IL-10R2) to get into the cells (Ank and Paludan, 2009; Kotenko, 2011). Similar to type I IFNs, stimulation by virus infection or TLR agonist induces type III IFNs (Kotenko et al., 2003; Coccia et al., 2004; Spann et al., 2004). Of note, Donnelly et al. found that the gene encoding the mouse ortholog of human IFNL1 contains a stop codon in the region of exon 1 and lacks the entire exon 2. Therefore, the gene Ifnl1 in mice does not encode a functional IFN-λ1 protein (Donnelly and Kotenko, 2010).

Ku70 and Ku80, proteins with molecular weight (MW) of 70 KDa and 80 KDa, respectively, are the essential components in the non-homologous end-joining (NHEJ) pathway. They are first identified in humans (Mimori et al., 1981). Ku70 is encoded by the X-ray repair cross-complementing protein (*XRCC*) 6 gene located on chromosome 22, and Ku80 is encoded by the *XRCC5* gene on chromosome 2. Hetero dimerization of Ku70 and Ku80 is essential for the stability of each protein. The lacking of one subunit leads to dramatically decreased intracellular level of the other subunit, suggesting that most Ku70 and Ku80 exist as a heterodimer (Nussenzweig et al., 1996; Gu et al., 1997). Such functional homologs have been identified in some prokaryotic lineages and almost all eukaryotes, including vertebrates, insects, and fungi (Dynan and Yoo, 1998; Aravind and Koonin, 2001; Bowater and Doherty, 2006). The Ku70/Ku80 heterodimer (so-called Ku) and a catalytic kinase subunit (DNA-PKcs) are often referred to as the subunit of the DNA-dependent protein kinase (DNA-PK) complex, which assembles in response to DNA double-strand breaks to repair the damaged DNA *via* NHEJ pathway (Fell and Schild-Poulter, 2015). The region between residues 439–592 at Ku80 C-terminus interacts with DNA-PKcs (Gell and Jackson, 1999; Singleton et al., 1999; Davis et al., 2013) and promotes the autophosphorylation of DNA-PKcs at DNA double-stranded breaks.

**Figure 1:**
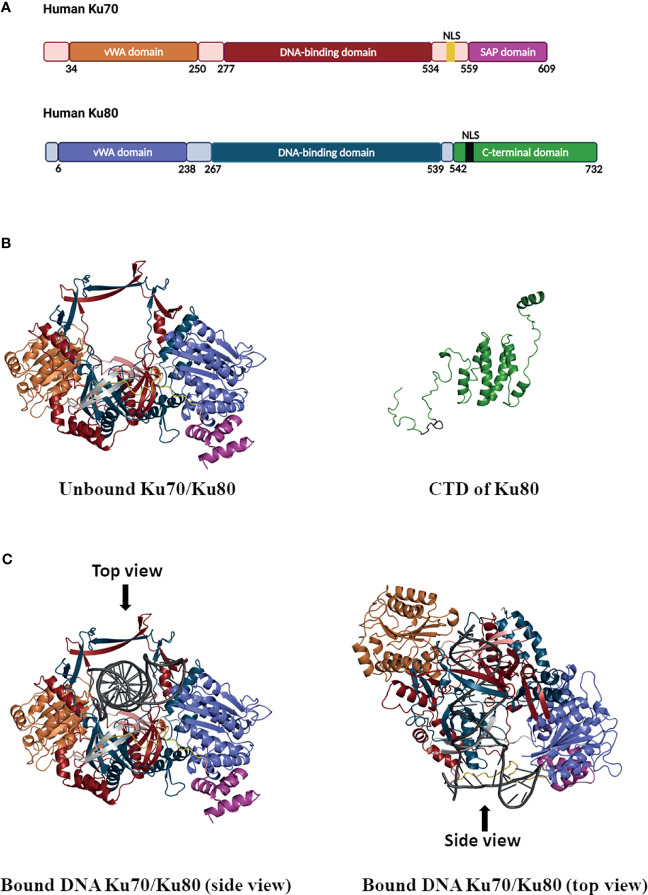
Schematic of Ku70/80 heterodimer domains and ribbon diagrams. **(A)** Domains in Ku70 and Ku80. In Ku70, the vWA domain is colored in orange, the DNA-binding domain is colored in firebrick, the SAP domain is colored in pink, the nuclear localization sequence (NLS: 539–556) is colored in yellow, and other parts are colored in light pink. In Ku80, the vWA domain is colored in purple, the DNA-binding domain is colored in blue, the c-terminal domain of Ku80 is colored in green, the NLS (561-569) of Ku80 is colored in black, and other parts are colored in light grey. **(B)** Unbound Ku70/Ku80 heterodimer with a view of Ku70 NLS (yellow) in the front (left panel). The range in the Ku70 model is from 35–609 amino acids, where the first 34 residues in the N-terminal domain (NTD) are truncated. The range in the Ku80 model is from 6–541 amino acids, where the first 5 residues in the NTD and residues 542–732 in the C-terminal domain (CTD) are truncated. The CTD domain of Ku80 is colored in green, and the corresponding NLS (561–569) is colored in black (right panel). **(C)** The structure model of the Ku70/Ku80 heterodimer bound with DNA. The left and the right panel demonstrate the side and top view, respectively, and bound DNA is colored in dark grey.

**Figure 2:**
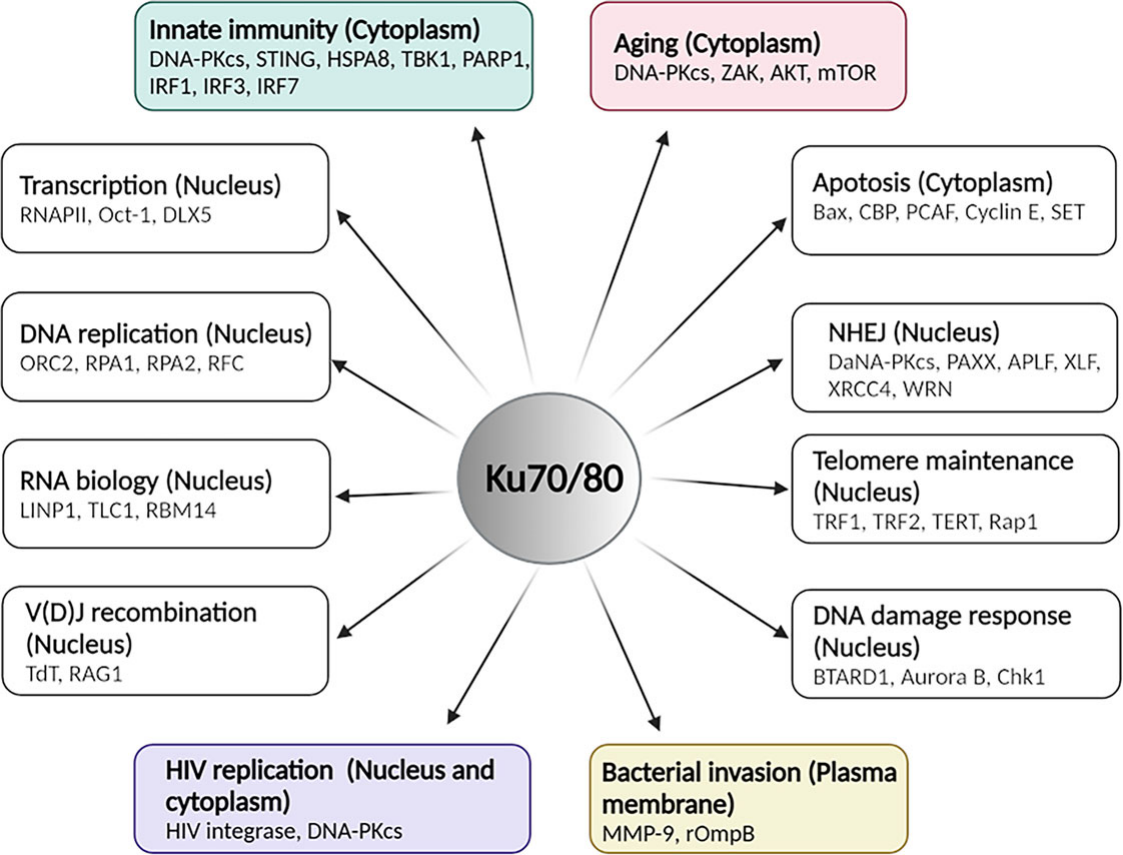
The overview of functions of Ku70/80 heterodimer in various cellular contexts. Ku70/80’s involvement in multiple cellular activities with indicated cellular localization and critical mediators. This illustration was created by using BioRender. The figure was adapted from the 2021 review by Abbasi et al. (Abbasi et al., 2021).

**Figure 3:**
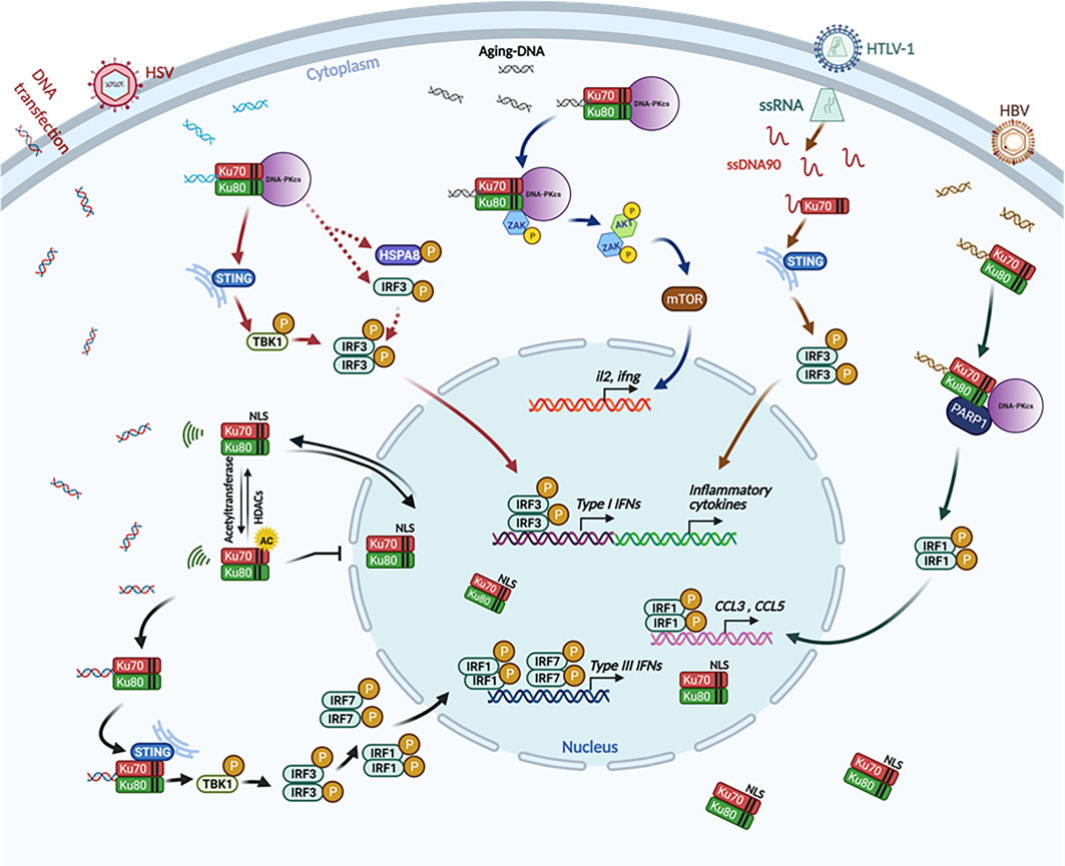
The involvement of Ku70 as a cytosolic DNA sensor to activate the innate immune response. Ku70 is identified as a cytosolic DNA sensor that induces type III IFNs through a STING-TBK1-IRF3, IRF1, and IRF7 signaling pathway. In this signaling pathway, cytoplasmic translocation of Ku70 is an initial and essential step (black arrows) (Zhang et al., 2011a; Sui et al., 2017; Sui et al., 2021); Ku70 and Ku80, together with DNA-PKcs (DNA-PK), are also involved in a STING-dependent (Ferguson et al., 2012) (solid arrow) and STING-independent (Burleigh et al., 2020) (dashed arrow) pathway to induce type I IFNs (red arrows). Ku70 is reported to sense HTLV-1 transcription intermediate product ssDNA90 and interacts with STING to induce IFNs and inflammatory cytokines, thereby modulating HTLV-1 replication (Wang et al., 2017) (brown arrows). The Ku70/80 heterodimer recognizes HBV-infection-derived DNA, then activates DNA-PKcs and PARP1 to induce CCL3 and CCL5 inflammatory cytokines (Li et al., 2016b) (green arrows). DNA-PK (the complex of DNA-PKcs, Ku70, and Ku80) senses aging-related cytoplasmic DNA in CD4+ T cells. This DNA sensing then induces T-cell proliferation and activation, as well as autoimmunity through the ZAK-AKT-mTOR pathway (Wang et al., 2021) (blue arrows). This illustration was created by using BioRender.

**Figure 4:**
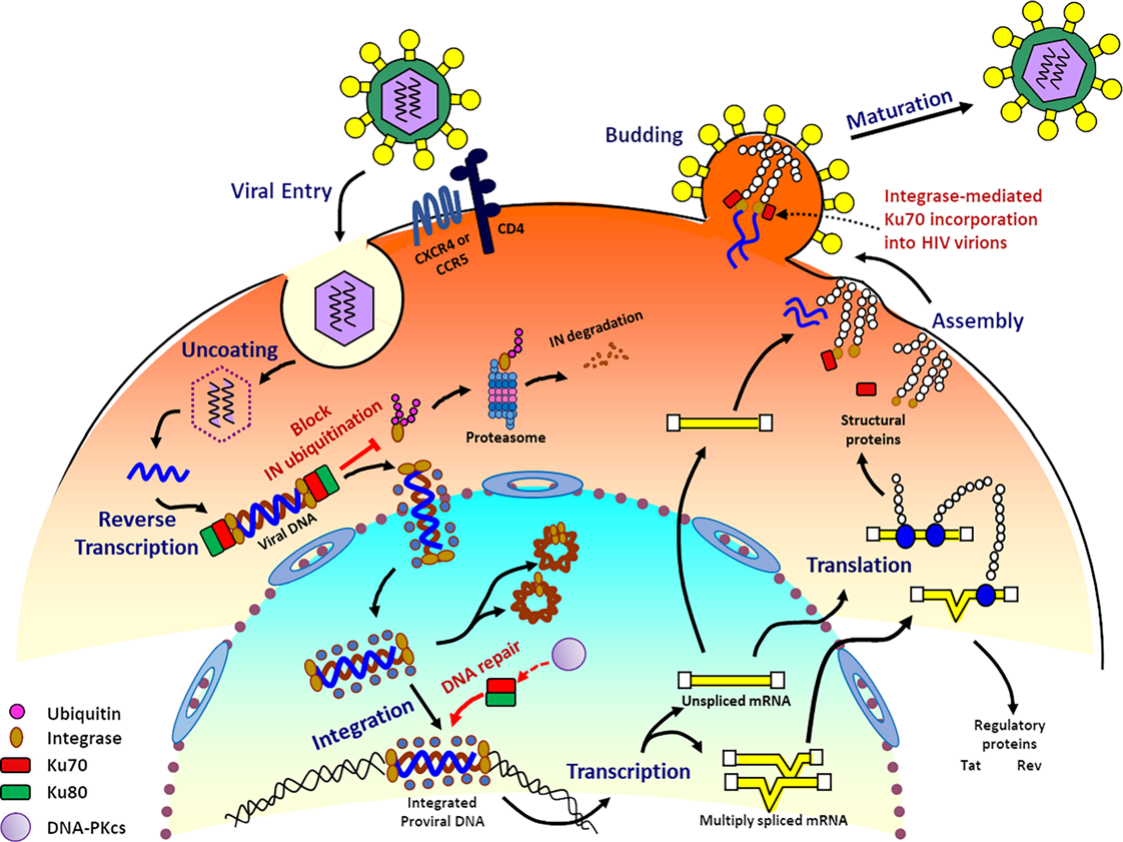
Ku70 is an indispensable host cellular factor in the early and late stages of the HIV-1 replication cycle. The interaction of IN with Ku70 during HIV reverse transcription prevents IN from degradation by the K48-linked Ub proteasome pathway. The interaction between Ku70 and IN decreases the modification level of IN by Ub in the cells. During the integration step, the initial binding of Ku70 and HIV-1 IN facilitates the recruitment of other members of the DNA-PK complex to the post-integration site. Then Ku70 serves as a member of DNA-PK and participates in the DNA gaps repair process through the NHEJ pathway, thereby completing the integration of viral DNA into the cell genome and enabling the HIV-1 viral replication. Ku70 is also packaged into HIV particles as early as its assembly stage and becomes part of HIV virions, and this process is mediated by HIV IN.

Ku70 and Ku80 are predominantly observed in the nucleus (Koike, 2002). Following translation of each protein in the cytosol, the Ku subunits can translocate from the cytoplasm into the nucleus together (Koike, 2002), or independently (Koike et al., 1999a), since each subunit possesses its own nuclear localization signal (NLS) (Koike et al., 2000). However, further functional studies have reported that Ku70 is not only involved in nuclear activities like DNA repair, transcription, and replication but is also involved in multiple cytosolic activities. Bax, a cytoplasmic protein, has been discovered to interact with Ku70, and this Ku70-Bax binding is indicated to inhibit Bax-mediated apoptosis (Cohen et al., 2004). In addition, many studies have implicated that cytosolic Ku has been shown to serve as a PRR that recognizes viral DNA in human cells and then induces type I and type III interferons or proinflammatory cytokines (Zhang et al., 2011a; Ferguson et al., 2012; Li et al., 2016b; Sui et al., 2017; Wang et al., 2017; Burleigh et al., 2020; Sui et al., 2021; Wang et al., 2021). This article summarized and discussed the Ku70-mediated innate immune response in detail and then highlighted potential strategies to modulate the innate immune cascades. The homology modeling for Ku70 and Ku80 is illustrated in **Figure 1**, the diverse functions of Ku70 are listed in **Figure 2**. The studies for Ku70 related to innate immunity are summarized in **Table 1** and illustrated in **Figure 3**. Beyond the role of Ku70 in innate immunity, roles of Ku70 in viral life cycle of Human Immune deficiency virus (HIV) (**Figure 4**) and in bacterial pathogen invasion are reviewed and discussed.

**Table 1:** List of studies about Ku70-involved innate immune response.

Sensor proteins	The source of nucleotides	Host cells	Signaling pathway	Induced cytokines	*In vivo*	References
Ku70	Plasmid DNA, bacterial DNA. HSV-2G, HSV-1	HEK, RD, THP-1, macrophages	STING-TBK1-IRF3, IRF1, and IRF7 pathway	IFN-λ1	N/A	(Zhang et al., 2011a; Sui et al., 2017; Sui et al., 2021)
DNA-PK	VACV, E. coli, ISD, HSV-1, MVA	Fibroblasts, MEF	STING-TBK1-IRF3	IFN-β, CXCL10, IL-6	Mice	(Ferguson et al., 2012; Peters et al., 2013; Scutts et al., 2018)
Ku70	pAAV-HBV plasmid, HBV	Liver-derived cells: Sk-Hep-1, Hep G2, Huh7, primary HSECs	DNA-PKcs and PARP1-IRF1	CCL3, CCL5	HBV-infected human patients	(Li et al., 2016b)
Ku70	HTLV-1 RTI ssDNA90	HeLa cells, PMA-THP-1	STING-TBK1-IRF3	IFN-β, IFN-λ, and TNF-α	N/A	(Wang et al., 2017)
DNA-PK	CT DNA	Human U937 cells, primary human hepatocytes, human fibroblasts	HSPA8-IRF3 (STING-independent sensing pathway)	IFN-β	N/A	(Burleigh et al., 2020)
Ku70/Ku80	ISD	Jurkat T cells, aged CD4+ T cells	ZAK-AKT-mTOR	IL-2, IFN-γ, T-cell proliferation	Mice	(Wang et al., 2021)

## Structure and Diverse Functions of Ku70 and Ku80

Ku70 forms a heterodimer with Ku80. Homology modeling for human Ku70 and Ku80, the proteins alone, and the complex with an oligo DNA substrate are shown in **Figure 1**. The entire structure of Ku70/Ku80 was not crystallized (Walker et al., 2001) due to the difficult experimental conditions of solving regions such as the Ku70 NLS. The homology modeling technique was used here to predict such missing segments. The hetero-oligomeric modeling pipeline implemented in SWISS-MODEL (Biasini et al., 2014) was used to predict the Ku70/Ku80 dimer structure. The target sequences of Ku70 (XRCC6, UniProt: P12956) and Ku80 (XRCC5, UniProt: P13010) were used as the input, and the crystal structure (PDB ID: 1JEQ (Walker et al., 2001)) was retrieved as the template for the final modeling. The target modeling segments show high sequence identities with their respective templates when aligned with those templates (95% for Ku70 and 97% for Ku80). Finally, the Ku70 structure was modeled, including 575 amino acids with the first 34 amino acids truncated, while the Ku80 structure with amino acids 6–541 was modeled. To create the C-terminal domain of the Ku80 model, we used the PDB ID of 6ZHE (Chaplin et al., 2021) as the template due to its relative completeness in this domain (**Figure 1B**). For incorporating the DNA coordinate into the Ku70/Ku80 model (**Figure 1C**), the PDB structure of 1JEY (Walker et al., 2001) was used as the anchor for fitting the model. Both the Ku70 and Ku80 protein possess a three-domain topology, including an N-terminal vWA (von Willebrand factor A) domain, a DNA-binding domain, and a C-terminal arm (Walker et al., 2001) (**Figure 1A**). The homology modeling of Ku heterodimer suggested a quasi-symmetric basket-like molecule with a narrow-preformed ring, which facilitates the binding of DNA to Ku (Walker et al., 2001) (**Figures 1B, C**).

The N-terminal vWA domains of Ku70 or Ku80 are composed of a six-stranded β-sheet in a Rossman fold (Walker et al., 2001). Disrupting the vWA domains in yeast Ku70/80 has been found to impair the function of Ku in DNA repair and telomere regulation (Ribes-Zamora et al., 2007). Although the amino edge of the vWA domain locates close to the DNA-binding groove, the vWA domain is not required for DNA binding. However, the Ku vWA domains may facilitate protein-protein interactions. For instance, the vWA domain of Ku80 has been found to interact with APLF, an NHEJ repair protein important for recruiting other repair factors (Grundy et al., 2013). So, the N-terminal vWA domains have minimal contribution to heterodimerization or DNA binding but are potentially involved in protein-protein interactions.

Meanwhile, the middle domain consists of a seven-stranded anti-parallel β-barrel and plays an essential role in Ku DNA binding and heterodimerization (Walker et al., 2001). Heterodimerization leads to a positively charged DNA-binding ring that fits sterically around the minor and major DNA grooves. Ku threads inwards on DNA like a nut threaded onto a bolt, with Ku70 positioned close and Ku80 far away to the DNA end (Yoo et al., 1999; Doherty and Jackson, 2001; Abbasi et al., 2021). Ku binds to dsDNA ends, 5′ and 3′ overhangs, or blunt ends with a higher binding affinity (Kd = 10^–9^ M). And it has a much lower binding affinity with circular DNA or the ends of single-stranded DNA (ss DNA) (Fell and Schild-Poulter, 2015).

C terminal regions of Ku contain a flexible linker and a globular structural domain (**Figure 1**). The C-terminal region of Ku70 contains a SAP (SAF-A/B, Acinus, and PIAS) domain encoded by residues 559–609 (Walker et al., 2001). Studies on other SAP domain proteins have implicated that SAP domains can bind DNA (Göhring et al., 1997; Suzuki et al., 2009). Using a pair of even shorter versions of Ku70, the Ku70_251-438 and Ku70_439-609 truncated mutants, Anisenko et al. have determined that the dsDNA is bound within the C-terminal part of the protein containing SAP domain (Anisenko et al., 2017b). While DNA binding to Ku, the SAP domain undergoes displacement, making itself close to the DNA-binding region of the Ku heterodimer (Rivera-Calzada et al., 2007; Makowski et al., 2016). Even the exact function of Ku70-SAP has not been completely investigated yet, the helical C-terminal arms of Ku contribute to heterodimerization and stabilize the interaction of Ku to DNA (Walker et al., 2001; Keijzers, 2018).

Notably, Ku70 and Ku80 per se possess an NLS in the molecule (as shown in **Figure 1** with Ku70 NLS in yellow and Ku80 NLS in black). The various NLS are classified into two types based on the pattern of molecular sequences: (1) a single cluster of basic amino acids, such as the NLS of the SV40 T-antigen, and (2) a bipartite type in which two basic amino acid regions are separated by a stretch of approximately 10 non-basic amino acids (Görlich and Mattaj, 1996). The Ku70 NLS belongs to type 2, and the sequence of Ku70 NLS is highly conserved among human, mouse, rat, hamster, and chicken (Koike et al., 1999b). While importing into the nucleus of the cells, the Ku70 NLS is recognized by the nuclear targeting complex, PTAC58, and PTAC97 (Koike et al., 1999b). Given that Ku70 is an NLS-possessing protein, it has been found predominately in the nucleus of unstimulated cells, such as HeLa, HEK (human embryonic kidney cells), and rhabdomyosarcoma (RD) cells (Sui et al., 2021).

Consistent with the illustrated structure, many studies have suggested that Ku possesses unusual DNA-binding properties, binding potently to the ends of dsDNA molecules in a sequence-independent manner. The unusual end-binding properties are required for various nuclear processes, such as NHEJ DNA repair (Critchlow and Jackson, 1998; Dobbs et al., 2010; Radhakrishnan et al., 2014; Menon and Povirk, 2016; Scully et al., 2019), V(D)J recombination of immunoglobin (Jackson and Jeggo, 1995; Fugmann et al., 2000; Bassing et al., 2002), telomerase maintenance (Bertuch and Lundblad, 2003; Indiviglio and Bertuch, 2009; Wood et al., 2015; Shay and Wright, 2019; Sui et al., 2020), transcription (Li et al., 1995; Giffin et al., 1996; Ono et al., 1996; Giffin et al., 1997; Dynan and Yoo, 1998; Mo and Dynan, 2002; Bunch et al., 2015), DNA damage response (Wang et al., 2000; Zhou and Elledge, 2000; Rouse and Jackson, 2002; Harper and Elledge, 2007; Jackson and Bartek, 2009; Fell and Schild-Poulter, 2012; Nowsheen and Yang, 2012; Blackford and Jackson, 2017), RNA biology (Yoo and Dynan, 1998; Peterson et al., 2001; Stellwagen et al., 2003; Ting et al., 2005; Pfingsten et al., 2012; Lamaa et al., 2016; Zhang et al., 2016b; Dutertre and Vagner, 2017; Shao et al., 2020; Thapar et al., 2020), and DNA replication (Barnes and Rio, 1997; Shao et al., 1999; Novac et al., 2001; Cosgrove et al., 2002; Matheos et al., 2002; Schild-Poulter et al., 2003; Park et al., 2004; Rampakakis et al., 2008; Miyoshi et al., 2009; Foster et al., 2011; Abdelbaqi et al., 2013; Sánchez and Russell, 2015; Teixeira-Silva et al., 2017). Such unusual DNA-binding properties also facilitate Ku70’s activities in the cytoplasm of the cells. Those activities include participating in Bax-mediated apoptosis (Cohen et al., 2004; Gomez et al., 2007; Mazumder et al., 2007; Kim et al., 2014) and serving as a cytosolic DNA sensor to activate the DNA-mediated innate immune response. An overview of Ku70/80 heterodimer functions in the various cellular processes is illustrated in **Figure 2**. In the following paragraphs, we will discuss roles of Ku70 in innate immunity, aging-related cytoplasmic DNA sensing, HIV replication and bacterial invasion in detail.

## Ku70 is Identified as a Novel Cytosolic DNA Sensor That Mediates Innate Immune Responses

Our lab previously reported, for the first time, that Ku70 is a novel DNA sensor to induce expression of IFN-λ1 rather than that of type-I IFNs (Zhang et al., 2011a). Plasmid DNA transfection or DNA virus infection-mediated IFN-λ1 induction was detected in HEK cells, RD cells, monocyte-derived macrophages, immature dendric cells, and—with a much lesser level—HeLa cells (Zhang et al., 2011a). These results indicated that the Ku70-mediated IFN-λ1 induction is consistently presented in multiple cell types.

Different forms of DNA transfection (e.g., single-stranded DNA, fragmented human genomic DNA, and bacterial DNA) and infection of DNA virus induce IFN-λ1 (Zhang et al., 2011a); IFN-λ1 mRNA was induced by both supercoiled or linearized forms of DNA plasmids. However, the linearized plasmid DNA significantly enhanced activation. This result was consistently supported by the property of Ku, which detects the end structure of DNA. Zhang et al. confirmed that over 500 bp of DNA triggers IFN-λ1 induction with the dependency of DNA length. By contrast, the production of IFN-λ1 was not detected with the transfection of DNA that was only 50 bp in length (Zhang et al., 2011a). One study demonstrated that titration of Ku to a fixed amount of linear dsDNA fragments produced ladders of shifted bands, which are proportional to the length of DNA. This data implicated that many Ku heterodimers bind to multiple sites on one dsDNA in a sequence-independent pattern (Blier et al., 1993). Based on those Ku properties, it was apparent that Ku70 induces activation of IFN-λ1 and that Ku70 recognizes intracellular DNA by DNA transfection or infection with a DNA virus, such as herpes simplex virus (HSV) type 1 (HSV-1) or type 2 (HSV-2), without any restriction in structure or sequence (Zhang et al., 2011a).

In addition to the fact that Ku70 senses DNA to induce type III IFNs, subsequent other studies indicated that Ku70 perse, or Ku70 in Ku70/Ku80 heterodimer, or Ku70 in the DNA-PK complex involves in the induction of type I IFNs and other inflammatory cytokines directly or indirectly. For example, Ku70 has also been reported to detect human T-lymphotropic virus type 1 (HTLV-1) reverse intermediate product ssDNA90 and induce IFN-α, IFN-β, IFN-λ, and RANTES (Wang et al., 2017). Additionally, the Ku70/Ku80 heterodimer senses the *in vitro* adenovirus-delivered hepatitis B virus (HBV) DNA and induces CCL3 and CCL5, thereby implicating that Ku70 modulates HBV replication (Li et al., 2016b). More interestingly, a recent study suggested that the Ku70/80 complex senses cytoplasmic DNA in aged CD4^+^ T cells and that this detection potentiated T-cell activation and aging-related autoimmune responses (Wang et al., 2021). Furthermore, Ferguson et al. reported that DNA-PK, a heterotrimeric protein complex composed of Ku70, Ku80, and DNA-PKcs, is able to activate downstream STING-TBK1-IRF3 signaling pathway when it recognizes foreign DNA (Ferguson et al., 2012). It has been further demonstrated that DNA-PK co-localizes with vaccinia virus (VACV) DNA during VACV infection. Virus infection-mediated IFN response is aborted when the components of DNA-PK were knocked-out (Ferguson et al., 2012).

Ku70, Ku protein, or DNA-PK have been implicated in having a role in sensing a variety of DNA or DNA viruses without restrictions. More importantly, many other DNA sensors, such as cGAS, require binding of double-stranded DNA to activate the sensor protein: a conformation change, thereby activating downstream signaling (Cai et al., 2014; Dempsey and Bowie, 2015; Xia et al., 2016). However, the Ku protein or DNA-PK complex does not have such conformational restriction; therefore, the Ku/DNA-PK-mediated innate immune response may become a perfect complementary pathway in the host defense system when other DNA-sensing pathways are impaired.

## The Downstream Signaling Pathway of Ku70

Many studies have indicated that Ku70, as a cytosolic DNA sensor, binds with DNA and mediates the downstream signaling pathway. However, “What is the adapter at the downstream signaling of Ku70?” was the next question. In Ku70-mediated type III IFN response, an investigation was initiated from the observation of IFN-λ1 induction in HEK and 293T (SV40-T antigen transformed HEK cell line) cells with GFP-encoding DNA plasmid transfection. With a similar green fluorescence signal observed between HEK 293 cells and 293T cells, DNA-induced IFN-λ1 induction was detected in HEK 293 cells but not in 293T cells. By comparing the expression level among different signal mediators associated with the cytosolic sensor, we found that the stimulator of interferon genes (STING) is not endogenously expressed in 293T cells. The gain-of-function and loss-of-function study confirmed the hypothesis that STING is the downstream adaptor of Ku70 to activate the IFN-λ1 signaling pathway. The co-immunoprecipitation assay further illustrated that Ku70 interacts with STING in the cytoplasm and forms a complex upon DNA stimulation (Sui et al., 2017). At this point, the activating pathway is quite similar to the DNA-PK-mediated STING-dependent pathway. DNA-PK was reported as a DNA cytosolic sensor to induce IFN-α or IFN-β (Ferguson et al., 2012). However, the interaction between DNA-PK and the downstream STING is in a transient pattern. After binding at three hours after DNA stimulation, STING dissociates from the complex, and this dissociation activates downstream signaling (Ferguson et al., 2012). STING is also the downstream target of Ku70 in sensing HTLV-1 intermediate product ssDNA90 and, therefore, induces type I interferons and inflammatory cytokines through phosphorylation of IRF3 (Wang et al., 2017).

In addition to STING as the downstream adaptor of Ku70 or DNA-PK, other proteins, namely DNA-PKcs and PARP1, are also reported as the adaptor proteins to Ku70/80 in sensing HBV DNA (Li et al., 2016b). While sensing aging-related DNA cytoplasmic accumulation, DNA-PK interacts with ZAK, AKT, and mTOR, inducing T-cell proliferation and aging-related autoimmunity (Wang et al., 2021). Another study recently claimed that DNA-PK is a potent sensor that activates the innate immune response with STING-independent signaling pathway. However, this pathway only exists in human cells and is not present in mouse cells (Burleigh et al., 2020). In this pathway, HSPA8/HSC70 is the adaptor protein for inducible phosphorylation and then activates downstream innate immune signaling (Burleigh et al., 2020). All those different Ku70-involved mechanisms determine the diverse patterns of innate immune response in a cell-type-dependent pattern. The coexistence of various molecular mechanisms is always an interesting topic in the research field of innate immunity.

Compared with the induction of type I IFNs, the kinetics of Ku70-mediated IFN-λ1 induction indicates a delayed induction profile. The IFN-λ1 mRNA expression is initiated at about 12 hours after DNA transfection. A profound protein level of IFN-λ1 can be detected at 48 hours after DNA transfection (Sui et al., 2017). As we know, cGAS- or IFI16-mediated innate immune response is usually induced as an earlier event after stimulation: for example, at six hours after stimulation (Unterholzner et al., 2010; Sun et al., 2013; Cai et al., 2014). The activation of downstream of cGAS or IFI16 is the STING-TBK1-IRF3 signaling pathway. IRF3 is endogenously expressed in most cells. The activation of IRF3 is detected at three hours after stimulation, indicating that IRF3 facilitates the induction as a faster and earlier event. By contrast, Ku70-mediated IFN-λ1 induction relies on activating the STING-TBK1-IRF3, IRF1, and IRF7 axis (Zhang et al., 2011a; Sui et al., 2017). IRF3 is activated first to produce a profound expression of IRF1 and IRF7, since IRF1 and IRF7 are not endogenously expressed in the cells. Once IRF1 and IRF7 are produced, IFN-λ1 and then can be significantly induced. In summary, similar to other DNA sensor-mediated innate immune responses, the kinetics of Ku70 involved innate immune response depends on the specific signaling pathway by which interferon or inflammatory cytokines are produced.

## The Cytoplasmic Translocation of Ku70 Is The Initial Step For Ku70 as a Cytosolic DNA Sensor

Ku70 was initially characterized as a DNA repair protein; its primary function serves in the nucleus. However, more and more studies have identified Ku70 as a cytosolic DNA sensor that mediates innate immune response. The downstream adaptor STING and another protein, HSPA8, were all found in the cells’ cytoplasm (Ferguson et al., 2012; Sui et al., 2017; Burleigh et al., 2020). So how can a nuclear protein sense cytosolic DNA and thereby initiate a downstream signaling pathway? This question led to identify the molecular mechanism at an earlier time point. In the case of Ku70-mediated IFN-λ1 induction by DNA transfection, Ku70 was observed predominately located in the cytoplasm of the cells, thereby facilitating the interaction between STING and Ku70 (Sui et al., 2017). So, it was speculated that upon DNA stimulation, Ku70 translocates from the nucleus to the cytoplasm of the cells. And this process is closely correlated with the induction of IFN-λ1.

The subsequent study using confocal microscopy confirmed that the cytoplasmic translocation of Ku70 is observed in the cells in which IFN-λ1 is induced by DNA transfection, such as HEK and RD cells. And consistently, such cytoplasmic translocation of Ku70 is not observed in HeLa cells, and similarly, DNA transfection does not induce IFN-λ1 induction in HeLa cells (Sui et al., 2021). In addition to DNA plasmid transfection, HSV-1, a DNA virus infection, also triggered the cytoplasmic translocation in HEK cells with a virus-strain-dependent manner. Ku70 cytoplasmic translocation and IFN-λ1 induction only in HEK cells infected with the HSV-1 McKrae strain. Those results further emphasized that the cytoplasmic translocation of Ku70 is a required step for Ku70-mediated IFN-λ1 induction (Sui et al., 2021). A quantification analysis with Western blot using cytosolic fractions was adapted to characterize the accumulation kinetics of cytoplasmic Ku70. The data demonstrated that the cytoplasmic translocation of Ku70 was started right after DNA stimulation and obtained the highest level at six hours after transfection, and then the accumulation of cytoplasmic Ku70 returned to a similar level as that in unstimulated cells (Sui et al., 2021). These data testified two points. First, the cytoplasmic translocation of Ku70 is a kinetics process. Ku70 translocates freely from the nucleus to the cytoplasm or from the cytoplasm back to the nucleus. How DNA transfection triggers the translocation remains unclear, but we hypothesized it is due to a change in a dynamic balance between the accumulation level of Ku70 in the nuclear and the cytoplasm. When cytosolic Ku70 recognizes and associates with cytoplasmic DNA, such interaction may interrupt the equilibrium between the cytosolic and the nuclear Ku70 and then drive the translocation of Ku70 from the nucleus to the cytoplasm. Second, the kinetic study further demonstrated that the translocation of Ku70 from the nucleus to the cytoplasm is an initial and essential step in the DNA-mediated IFN-λ1 innate immune response. Compared with the time course of IFN-λ1 induction, the translocation of Ku70 occurred one hour right after DNA transfection and peaked at six hours. All this happened before IFN-λ1 induction. Consequently, it is reasonable to speculate that the translocation of Ku70 happened first and that IFN-λ1 induction occurred later since we further confirmed that recombinant IFN-λ1 does not induce the cytoplasmic translocation of Ku70 (Sui et al., 2021). Like Ku70, IFI16, another DNA sensor protein, has been reported to recognize and sense DNA not only in the cytoplasm but also in the nucleus of the cells, and its sensing capabilities depend on the distribution of IFI16 (Li et al., 2012; Dell’oste et al., 2014; Ansari et al., 2015). IFI16 detects and binds to herpes viral DNA in the nucleus of the cells. However, detection of transfected DNA or cytoplasmic viral DNA occurs in the cytoplasm. Ku70 ubiquitously expresses in the nucleus as a nuclear protein; however, there is no evidence to indicate that Ku70 can also serve as a nuclear DNA sensor protein.

In addition to our detailed study about the cytoplasmic translocation of Ku70, Li et al. also reported that the cytoplasmic-translocated Ku70/Ku80 complex senses HBV DNA and then induces hepatitis-associated chemokine secretion (Li et al., 2016b). This kind of nuclear-cytoplasmic (N-C) trafficking has become a conventional mechanism for these multifunctional DNA sensors. As we know, cGAS recognizes cytosolic DNA. This detection produces the second messenger 2’3’-cGAMP, and the cGAMP in turn initiates STING-dependent downstream signaling to induce type I IFNs. However, more recently, Sun et al. demonstrated that cGAS is located both in the cytoplasm and in the nucleus, and cGAS is required to export into the cytoplasm in response to DNA stimulation. (Sun et al., 2021). Therefore, the N-C trafficking is required for Ku70 and other multiple-functional proteins to conduct their cytosolic and nuclear activities.

## The Cooperative Pattern of Ku70, Ku80, and DNA-PKcs In Mediating Innate Immune Response

Ku70 is a subunit of the heterotrimeric protein complex DNA-PK composing of Ku80 and the catalytic subunit DNA-PKcs. While we identified Ku70 as a novel cytosolic DNA sensor that induces IFN-λ1 innate immune response (Zhang et al., 2011a; Sui et al., 2017; Sui et al., 2021), we hope to determine whether Ku80 or DNA-PKcs are also involved in the cytosolic-DNA-sensing activity.

It has been reported that DNA-PK serves as a PRR, recognizing cytoplasmic DNA and inducing the production of type I IFNs (Ferguson et al., 2012; Burleigh et al., 2020). The Ku heterodimer (Walker et al., 2001) and DNA-PKcs (Hammarsten and Chu, 1998) can directly bind to DNA; however, in the absence of either Ku70 or Ku80, the binding affinity of DNA-PKcs with DNA is dramatically decreased (Yaneva et al., 1997). These findings implicated that each subunit of the DNA-PK complex plays an essential role. Consistent with this study, we also observed the existence of Ku80, but not DNA-PKcs, in the complex of Ku70-STING (Sui et al., 2017). Additionally, we observed the co-localization of Ku80 with Ku70 in the nucleus of unstimulated cells and the cytoplasm of DNA-transfection-stimulated cells. Those data suggest that Ku80 is translocated with Ku70 from the nucleus to the cytoplasm (Sui et al., 2021).

However, we previously reported that DNA-mediated IFN-λ1 induction substantially decreased, when Ku70 is transiently knocked down; in contrast, knocking down of Ku80 has no impact on the induction of IFN-λ1. To further validate the role of Ku70 and Ku80 in DNA-mediated innate immune response, the IFN‐λ1 promoter reporter assay by overexpressing each subunit was utilized in the study. The result from the assay consistently demonstrated that overexpression of Ku70 highly activates the IFN‐λ1 promoter. However, the overexpression of Ku80 had no impact on IFN‐λ1 promoter activation. Moreover, the results of the co-immunoprecipitation assay directly exclude the presence of DNA-PKcs in the complex of Ku70-STING. Therefore, all those studies suggested that Ku80 and DNA-PKcs may not be directly involved in DNA-mediated IFN-λ1 induction (Zhang et al., 2011a; Sui et al., 2017).

In studies about DNA-PK as the cytosolic DNA sensor to induce the innate immune response, it seems that DNA-PKcs is the key factor to mediate downstream signaling and that the involvement of Ku70 or Ku80 enhances the sensing capability of DNA-PKcs (Ferguson et al., 2012; Burleigh et al., 2020). In HBV infection, the Ku70/80 complex senses infected HBV DNA, and DNA-PKcs and PARP1 act as a downstream adaptor to activate hepatitis-associated chemokine secretion (Li et al., 2016b).

Ku70-involved innate immune response shows various patterns for the participation and function of Ku70, Ku80, and DNA-PKcs. In general, like their function in the DNA repair process, they work together as a whole complex (Ferguson et al., 2012; Burleigh et al., 2020; Wang et al., 2021), but in the case of type-III IFN response and Ku70 sensing HTLV-1, the functional element is Ku70 itself. However, we have become aware that when Ku70 or Ku80 is expressed individually, neither of them are stable (Satoh et al., 1995) and that the absence of one of the subunits leads to a remarkable reduction in the stable level of the other one (Errami et al., 1996; Gu et al., 1997; Singleton et al., 1997). Consequently, it is hard to precisely elucidate the function of Ku70 or Ku80 alone by completely knocking out one or the other. Further study will help to illuminate the detailed molecular mechanism of how Ku70, Ku80, or DNA-PKcs cooperate and facilitate DNA-sensing activity.

## The Potential Regulation Factors Involved in Ku70-Mediated Innate Immune Response

Further studies have reported that Ku70, predominantly located in the nucleus of the cells, has a cytoplasmic translocation from the nucleus, then conducts its cytosolic activities, such as sensing invading cytosolic DNA to induce an innate immune response (Zhang et al., 2011a; Sui et al., 2017; Sui et al., 2021) or binding with invading viral elements/proteins to modulate virus replication (Li et al., 2016b). Therefore, abundant amounts of cytoplasmic protein accumulation seem to be essential for Ku70 to successfully recognize cytosolic DNA and activate the downstream IFN signaling pathway. Our observations, nuclear retention of Ku70, because of the treatment with leptomycin B, severely attenuates the IFN-λ1 response to DNA stimulation (Sui et al., 2021), indicating that cytoplasmic translocation is a critical factor for Ku70’s cytosolic DNA sensing.

Our group confirmed that acetylation at Ku70-NLS regulates the localization of Ku70 in the nucleus or in the cytoplasm, which is consistent with the finding from other groups (Fujimoto et al., 2018), and we first reported that acetylation modulates Ku70’s DNA‐sensing activity (Sui et al., 2021). While importing into the nucleus, Ku70 has to interact with the Impα/Impβ complex to facilitate nuclear translocation. With the acetylation at the region of Ku70-NLS, the interaction between acetylated Ku70 and the Impα/Impβ complex is severely decreased. Therefore, acetylated Ku70 is predominantly located in the cytoplasm of the cells (Fujimoto et al., 2018). In line with Fujimoto’s finding, we further demonstrated that acetylated Ku70 highly induces DNA-mediated IFN-λ1 induction (Sui et al., 2021).

The acetylation level of a protein depends on the dynamic balance between the activity of acetylation and deacetylation enzymes (Ansari et al., 2015; Gong et al., 2019). Multiple lysine residues have been identified as acetylation locations on Ku70 and Ku80 (Cohen et al., 2004; Subramanian et al., 2013; Al-Emam et al., 2014; Koike et al., 2017). Acetylation at Ku70 lysine residues, K539, K542 (Subramanian et al., 2005; Subramanian et al., 2013) and K317, K331, K338 (Al-Emam et al., 2014) impaired the function of Ku70 in NHEJ, since those lysine residues of Ku70 are required for Ku70 binding with dsDNA ends during NHEJ process. Two histone acetyltransferase enzymes, CBP and PCAF, are responsible for Ku acetylation (Cohen et al., 2004). Histone deacetylases (HDACs), a family of deacetylation enzymes, regulate the deacetylation of multiple non‐histone proteins and, therefore, impact functions by changing their activity, such as cellular localization and protein-protein interactions (Subramanian et al., 2005; Roger et al., 2011). More than 50 non‐histone proteins, including p53 and Ku70, have been defined as the substrates of HDACs (Chaudhary et al., 2014; Gong et al., 2019). Trichostatin A (TSA), an inhibitor sensitive to class I/II deacetylases, was utilized in our study to evaluate the impact of this deacetylase inhibitor on the Ku70 cytoplasmic accumulation and DNA-mediated IFN-λ1 induction. The data implicated that TSA treatment dose-dependently enhances the cytoplasmic accumulation of Ku70 and increases DNA-mediated IFN-λ1 induction. (Sui et al., 2021). As we demonstrated in our study, the relationship of the acetylation levels of Ku70 and DNA-mediated innate immune response may provide a simple and elegant strategy, modulating the acetylation levels of the target protein to regulate its localization-dependent activities.

Ku70 and Ku80 are generally believed to always form heterodimers. And it has been consistently confirmed in our previous study that Ku80 translocates from the nucleus to the cytoplasm together with Ku70. The confocal microscopy analysis indicated that Ku70 and Ku80 colocalized together in the nucleus of unstimulated cells, and then both translocate from the nucleus to the cytoplasm upon a DNA stimulation (Sui et al., 2021). However, Koik et al. demonstrated that the localization of Ku80 does not entirely coincide with that of Ku70, Ku80 protein was transported to the nucleus without heterodimerization with Ku70. The Ku80 NLS was demonstrated to be mediated to the nuclear rim by two components of PTAC58 and PTAC97. This findings support the idea that Ku80 can translocate to the nucleus using its own NLS independent of the translocation of Ku70 (Koike et al., 1999a). On the other hand, using the site-directed mutagenesis technique, the same group demonstrated that Ku70 can also translocate to the nucleus without heterodimerization with Ku80 or independent of DNA-PK autophosphorylation (Koike et al., 2000).

The N-C or C-N translocation of DNA-PKcs is rarely reported. We have confirmed DNA-PKcs is not involved in Ku70-mediated IFN-λ1 induction. Co-immunoprecipitation assay suggested DNA-PKcs is not present in the Ku70-STING complex. Therefore, implicating that DNA-PKcs does not translocate together with Ku70 or Ku80 from the nucleus to the cytoplasm upon a DNA transfection or DNA virus infection (Sui et al., 2017). Other factors may involve in facilitating the translocation of DNA-PKcs. Further study is required to help us understand the translocation of DNA-PKcs.

Regulation of the N-C translocation has been defined as an essential mechanism to control protein activities. Whether some other cellular factors may facilitate Ku70, Ku, or DNA-PK to respond to specific stimuli by regulating its nuclear or cytoplasmic localization remains to be identified. Better understanding the N-C translocation of Ku70, Ku80 and DNA-PKcs may provide unique insights into the multiple functions of Ku70 in the DNA repair process, Bax-mediated apoptosis, and innate immune response.

In addition to regulating the N-C transport, the detailed mechanisms regarding Ku70-mediated innate immunity offer multiple strategies to downregulate cytosolic DNA-induced autoimmunity or enhance innate immune response under the context of DNA vaccination. For example, Wang et al. found that the Ku70/Ku80 heterodimer recognizes aging-related DNA accumulation in the cytoplasm of human or mouse CD4^+^ T cells. The sensing by the Ku complex further recruits DNA-PKs on the site and triggers the phosphorylation of ZAK. Subsequently, it activates the AKT-mTOR signaling pathway, which enhances the proliferation of CD4^+^ T cells and accelerates the pathology progress of experimental autoimmune encephalomyelitis (EAE) in mice (Wang et al., 2021). Consequently, based on the discovered molecular mechanism, the group further developed an inhibitor specific against ZAK to dampen the pathology progress of EAE (Wang et al., 2021).

It is known that many viruses possess the system to escape from the innate immune response by host cells. The mechanism of the immune escape by a DNA virus, Vaccinia virus (VACV), has been investigated (Bowie and Unterholzner, 2008; Elde et al., 2012). The VACV C16 protein was reported as the first protein to inhibit DNA-PK-mediated signaling (Peters et al., 2013). It has been demonstrated that the C-terminal region of C16 binds directly to the Ku70/Ku80 complex, therefore blocking the sensing of Ku to DNA. The protein VACV C16 is not endogenously expressed on VACV strain Western Reserve. So the intranasal infection of this virus strain in mice leads to enhanced innate immune response and less symptoms of viral infection-related sickness (Fahy et al., 2008; Peters et al., 2013). Another protein of VACV, C4, is later identified targeting DNA-PK to inhibit DNA-PK-mediated signaling. VACV C4 possesses a similar sequence as C16, so it shares a similar mechanism to block DNA binding to DNA-PK by binding to the Ku complex. The absence of C4 promotes innate and adaptive immune responses (Scutts et al., 2018). Overall, these findings demonstrate that viral proteins help to evade the sensing of the viral genome by inhibiting the activity of PRR, therefore highlighting alternative strategies to regulate the innate immune response.

Similar to VACV, DNA virus HSV-1 has also shown the ability to evade innate immune responses in host cells (Su et al., 2016; Lum and Cristea, 2021). Studies from Zheng’s lab implicated that HSV-1 VP24, a serine protease, could also inhibit dsDNA-initiated IFN production by blocking the interaction between IRF3 and TBK1 and therefore dampening the phosphorylation of IRF3 (Zhang et al., 2016a). Another study demonstrated that HSV-1 VP16 could interrupt IRF3 recruiting the CREB-binding protein coactivator, thus inhibiting IRF3-mediated downstream signaling (Xing et al., 2013). Furthermore, US3 of HSV-1, another viral protein kinase, has been reported to prevent IRF3 activation and inhibit type-I IFN production by hyper phosphorylating IRF3 at Ser175 (Wang et al., 2013; Wang et al., 2014). It has been reported that HSV-1 ICP27 interacts with TBK1 and STING, which impairs the activation of downstream transcription factor IRF3. (Christensen et al., 2016). Our previous study also found that the Ku70-mediated type-III IFN response was induced in an HSV-1 strain-dependent manner: Infection with the HSV-1 McKrae strain triggers the cytoplasmic translocation of Ku70 and induces IFN-λ1 induction, while infection with the HSV-1 MacIntyre strain does not. Therefore, we speculated that the HSV-1 MacIntyre strain might encode specific viral proteins that may inhibit the signaling pathway of IFN induction. Further studies are needed to identify the specific molecular mechanism for HSV-1 immune evasion. As we listed above, all these observations implicated that HSV-1-encoded viral proteins to facilitate HSV-1 immune evasion could interrupt the downstream signaling of DNA-mediated signaling pathway, therefore providing potential strategies to regulate any signaling pathway with shared downstream signaling adaptors, such as STING, TBK1, and IRF3.

In summary, with an aim to highlight innate immune response mediated by DNA virus infection in a battle against viral infection, a better understanding of the interplay between host innate immune response and viral immune evasion would provide intriguing novel strategies to help develop diverse therapies to treat viral infection-related diseases.

## Beyond the Role of Ku70 In Innate Immunity: A Role of Ku70 in HIV Replication Cycle and Bacterial Internalization

HIV needs many cellular factors to facilitate its replication (Emig-Agius et al., 2014). Ku70 and Ku80 are reported as host partners involving in HIV replication (Waninger et al., 2004; Studamire and Goff, 2008; Santos et al., 2012; Schweitzer et al., 2013; Emig-Agius et al., 2014; Hultquist et al., 2016; Li et al., 2016a). Several studies found that Ku70/Ku80 heterodimer binds with HIV genomic RNA or TAR RNA at the 5’ end of mRNA transcripts. Those data further implicated that the Ku complex may regulate the transcription process of HIV. (Kaczmarski and Khan, 1993; Yoo and Dynan, 1998); the interactions between Ku and HIV RNA may also impact the transcription level of HIV and the latency property of HIV (Manic et al., 2013). Additionally, Ku could also regulate transcriptional elongation by interacting with the RNA hairpin structure of 7SK snRNA, a scaffold protein for forming the 7SK snRNP complex (Shadrina et al., 2016; Shadrina et al., 2020). Several contradictory studies also show that Ku involves in retroviral DNA integration (Daniel et al., 1999; Baekelandt et al., 2000; Daniel et al., 2004; Knyazhanskaya et al., 2016) in the transcription of integrated provirus (Jeanson and Mouscadet, 2002; Tyagi et al., 2011; Manic et al., 2013; Shadrina et al., 2016), and in functions of HIV-1 matrix protein (Li et al., 2016a). Another evidence demonstrated that the DNA-PK complex involves in the induction of apoptosis in activated CD4^+^ T cells at the early stage of HIV infection (Cooper et al., 2013).

HIV-1 integrase (IN) is an essential viral enzyme involving in several viral replication steps. Meanwhile, IN is also an unstable protein and degraded by the N-end rule pathway through the host ubiquitin-proteasome machinery (Mulder and Muesing, 2000). However, it remains unknown how HIV-1 IN is protected from degradation during HIV replication. Zheng et al. demonstrated that Ku70 from host cells interacts with HIV-1 IN and prevents it from the Lys48-linked polyubiquitination proteasomal pathway. Additionally, Ku70 can decrease the overall protein polyubiquitination level and specifically deubiquitinate IN by binding with HIV-1 IN (Zheng et al., 2011). Mutagenic studies by Anisenko et al. showed that the amino acid residues 51-160 of HIV-1 IN interacts with 251-438 aa of Ku70. It is further reported that the N-terminal region (1-250 aa) of Ku70 interacts with the α6-helix region located at the 200-220 residues of IN, and the single mutations at E212A or L213A abrogate the interaction. Those findings highlighted the essential role of the 200-220 aa residues of IN in forming a complex with Ku70 (Anisenko et al., 2017a).

Additionally, knockdown of Ku70 significantly inhibits the HIV-1 virus replication in virus-producing cell lines or HIV-infected CD4+ T cells, and the copy number of two-long terminal repeat (LTR) circles and integrated proviral DNA cannot be detected. Those data implicated that Ku70 is an indispensable factor at the early and the late stages of HIV-1 replication (Zheng et al., 2011) (as illustrated in **Figure 4**). HIV-1 IN is an essential enzyme in HIV virions and integrates the proviral DNA into the host genomic DNA. Integration is a critical step during HIV-1 replication. (Cherepanov et al., 2003; Faure et al., 2005; Passos et al., 2017). In detail, IN binds viral DNA and then catalyzes the cleavage of dinucleotides from both 3’-ends of viral DNA. The complex of 3’-processed viral DNA and IN helps recruit some other viral and cellular proteins as cofactors. Subsequently, the whole complex imports into the nucleus. The second step of integration happens in the nucleus of the host cells. IN inserts the processed viral DNA into one strand of the genomic DNA of host cells (Lesbats et al., 2016). This insertion leads to 5-nucleotide gaps (Vincent et al., 1990; Vink et al., 1990; Lesbats et al., 2016). As a result, 3’-ends of viral DNA are then covalently associated with the cellular DNA. However, an overhang is formed at the 5′-ends because of an unpaired dinucleotide (Knyazhanskaya et al., 2019). In order to complete the integration process, the intermediate product has to be repaired (Knyazhanskaya et al., 2016). Knyazhanskaya et al. proposed that the direct binding between Ku70 and HIV-1 IN greatly facilitates the recruitment of Ku80 and DNA-PKcs to the integration site. And then, the whole DNA-PK complex sufficiently functions in initiating the DNA repair process by the NHEJ pathway and resumes efficient HIV-1 replication (Knyazhanskaya et al., 2019).

Interestingly, Zheng et al. found that Ku70 is incorporated into HIV viral particles (Zheng et al., 2011). Nascent HIV viruses contain Gag and GagPol polyproteins, and viral genomic RNAs (as illustrated in **Figure 4**). The polyproteins are composed of several HIV proteins and immature forms of IN, located at the C’- terminus end (Imamichi et al., 2021). Thus, Ku70 maybe incorporated in the virion during assembly *via* the immature IN. As IN regulates viral maturation (Engelman et al., 1995; Bukovsky and Göttlinger, 1996; Balakrishnan et al., 2013; Hoyte et al., 2017; Imamichi et al., 2021). Further study may reveal more roles of Ku70 during retrovirus infection and replication.

Overall, many studies have provided examples of how HIV-1 viruses commandeer host cellular machinery to protect themselves and facilitate viral replication (Zheng et al., 2011). Consequently, identifying the host cell factors that participate in these processes and determining their functions in HIV viral replication may lead to discovering novel therapeutic targets to fight HIV (Adamson and Freed, 2010; Tintori et al., 2014). Ku70 may become an ideal therapeutic target to treat patients infected with multi-drug-resistant HIV variants.

As we discussed in the current review, cytosolic Ku70, which is translocated from the nucleus to the cytoplasm, can sense cytosolic DNA to induce innate immune response (Zhang et al., 2011a; Ferguson et al., 2012; Li et al., 2016b; Sui et al., 2017; Wang et al., 2017; Burleigh et al., 2020; Sui et al., 2021; Wang et al., 2021), and can inhibit Bax-mediated apoptosis (Sawada et al., 2003; Mazumder et al., 2007). Additionally, Ku70 has also been found localized in the plasma membrane, where it can interact with metalloprotease 9 (MMP-9) (Monferran et al., 2004b), fibronectin (Monferran et al., 2004a) and participate in heterologous and homologous cell adhesion (Koike, 2002). It was also reported that the cell-surfaced Ku70 acts as a receptor for the infection of *Rickettsia conorii* (*R. conorii*), a negative gamma bacterium; the rickettsial protein, rOmpB, binds to Ku70 as a ligand. The interaction plays an important role in initiating infection signals, ultimately leading to bacterial entry (Martinez et al., 2005). The plasma membrane-associated Ku70 has also been identified in lipid rafts, and so it has been speculated that the existence of Ku70 within these domains may play an essential role in pathogen entry and signal transduction (Lucero et al., 2003).

Beyond the role of Ku70 in innate immunity, those studies about the involvement of Ku70 in pathogen invasion and HIV replication highlighted a further understanding of the interplay between the host protein Ku70 and pathogen. Further investigation could lead to the development of novel, efficacious therapies in the treatment and prevention of infectious diseases.

## Conclusions and Perspectives

The study of Ku70/80 is expanding to encompass numerous research fields, including regulatory processes. More and more promising research emphasizes the role of Ku in innate immunity, the development of a small-molecule Ku inhibitor (Weterings et al., 2016), and the essential clinical relevance of Ku. Exploring the molecular mechanism by which the Ku- or DNA-PK-involved innate immune response confers various strategies to regulate innate immune cascade and could shed light on the role of Ku70 in autoimmune diseases, vaccine development, or aging-related abnormalities. Further investigation could lead to more discoveries at both the basic and translational research levels.

Delineation of the molecular mechanisms of Ku70-mediated innate immune response, especially the cytoplasmic translocation of Ku70, provides novel strategies to regulate innate immune cascades in response to the invasion of foreign microbe DNA or the accumulation of abnormal cellular DNA. Some autoimmune diseases are caused by the persistent induction of proinflammatory cytokines and IFNs. The emergence of mutations in some genes, including *Trex1*, *RNase H*, *SAMHD1*, and others (Crow et al., 2006a; Crow et al., 2006b; Rice et al., 2009; Crow et al., 2015) leads to the abnormal accumulation of cellular DNA. Those abnormal cytoplasmic DNAs serve as dangerous PAMPs and are recognized by potential PRRs in host cells, and then initiate continuous production of innate immune cytokines. Hypothetically, inhibition of the cytoplasmic translocation of DNA sensors, such as Ku70 and IFI16, with some small compounds is expected to abrogate the sensing of cytosolic DNA, therefore downregulating IFN response and providing effective interventions for these autoimmune diseases. Similar strategies may also be used to decrease the over-response of host cells to some viral infections (Sun et al., 2021).

Future research may reveal a more comprehensive understanding of the multiple roles of Ku70, especially in the field of Ku70-involved innate immune networks. These findings would help us solve some remaining questions: how Ku70 regulates its activities in the nucleus and the cytoplasm, and whether it is possible that Ku70 also serves as a nucleus DNA sensor like IFI16 (Kerur et al., 2011; Unterholzner and Bowie, 2011; Li et al., 2012; Dell’oste et al., 2014; Ansari et al., 2015; Roy et al., 2019). Overall, a better understanding of the multiple functions of Ku70 at both the cellular and organismal level would provide new insights into treatments of infectious diseases and autoimmune abnormalities.

## Author Contributions

HS and TI conceptualized the work and contributed to writing the manuscript. MH and WC contributed to homology modeling for human Ku70 and Ku80. All authors contributed to editing the manuscript and approved the submitted version.

## Funding

This research was supported (in part) by the National Institute of Allergy and Infectious Diseases. This project has been funded in whole or in part with federal funds from the National Cancer Institute, National Institutes of Health, under Contract No. HHSN261200800001E.

## Author Disclaimer

The content of this publication does not necessarily reflect the views or policies of the Department of Health and Human Services, nor does mention of trade names, commercial products, or organizations imply endorsement by the U.S. Government.

## Conflict of Interest

The authors declare that the research was conducted in the absence of any commercial or financial relationships that could be construed as a potential conflict of interest.

## Publisher’s Note

All claims expressed in this article are solely those of the authors and do not necessarily represent those of their affiliated organizations, or those of the publisher, the editors and the reviewers. Any product that may be evaluated in this article, or claim that may be made by its manufacturer, is not guaranteed or endorsed by the publisher.

## Abbreviations

DNA-PK, DNA-dependent protein kinase; NHEJ, non-homologous end-joining; PRRs, pattern recognition receptors; PAMPs, pathogen-associated molecular patterns; TLRs, Toll-like receptors; RIG-I, retinoic acid-inducible gene I; NF-κB, nuclear factor κB; IFN, interferon; IRF3, Interferon regulatory factor 3; IL-1β, interleukin-1βb; DDX41, DEAD-box helicase 41; IFI16, gamma-interferon-inducible protein; DAI, DNA-dependent activator of IFN-regulatory factors; dsDNAs, double-stranded DNAs; LRRFIP1, Leucine-rich repeat flightless-interacting protein 1; DHX, DEAH box protein; AIM2, absent in melanoma 2; PYHIN, pyrin- and HIN200-domain-containing protein; MyD88, myeloid differentiation primary response 88; cGAS, cyclic GMP-AMP synthase; ASC, apoptosis-associated speck-like protein; XRCC, X-ray repair cross-complementing protein; NLS, nuclear localization signal; RD, rhabdomyosarcoma; VACV, vaccinia virus; HBV, hepatitis B virus; HSV, herpes simplex virus; HDACs, histone deacetylases; TSA, trichostatin A; N–C, nuclear-cytoplasmic; EAE, experimental autoimmune encephalomyelitis; IN, integrase; Ub, ubiquitination; CT DNA, calf thymus sonicated DNA; HTLV-1, human T-lymphotropic virus type 1; NTD, N-terminal domain; CTD, C-terminal domain; ISD, interferon stimulatory DNA.
